# Sleeve Gastrectomy and Roux-en-Y Gastric Bypass Achieve Similar Early Improvements in Beta-cell Function in Obese Patients with Type 2 Diabetes

**DOI:** 10.1038/s41598-018-38283-y

**Published:** 2019-02-12

**Authors:** Jamie A. Mullally, Gerardo J. Febres, Marc Bessler, Judith Korner

**Affiliations:** 1 Department of Medicine, Columbia University College of Physicians and Surgeons, New York, NY 10032 USA; 2 Department of Surgery, Columbia University College of Physicians and Surgeons, New York, NY 10032 USA

## Abstract

Bariatric surgery is a treatment option for obese patients with type 2 diabetes mellitus (T2DM). Although sleeve gastrectomy (SG) is growing in favor, some randomized trials show less weight loss and HbA1c improvement compared with Roux-en-Y gastric bypass (RYGB). The study objective was to compare changes in beta-cell function with similar weight loss after SG and RYGB in obese patients with T2DM. Subjects undergoing SG or RYGB were studied with an intravenous glucose tolerance test before surgery and at 5–12% weight loss post-surgery. The primary endpoint was change in the disposition index (DI). Baseline BMI, HbA1c, and diabetes-duration were similar between groups. Mean total weight loss percent was similar (8.4% ± 0.4, p = 0.22) after a period of 21.0 ± 1.7 days. Changes in fasting glucose, acute insulin secretion (AIR), and insulin sensitivity (Si) were similar between groups. Both groups showed increases from baseline to post-surgery in DI (20.2 to 163.3, p = 0.03 for SG; 31.2 to 232.9, p = 0.02 for RYGB) with no significant difference in the change in DI between groups (p = 0.53). Short-term improvements in beta-cell function using an IVGTT were similar between SG and RYGB. It remains unclear if longer-term outcomes are better after RYGB due to greater weight loss and/or other factors.

## Introduction

Bariatric surgery is the most effective long-term treatment for obesity and is also considered a treatment option for type 2 diabetes mellitus^1^. The two most commonly performed bariatric surgeries are Roux-en-Y gastric bypass (RYGB) and laparoscopic sleeve gastrectomy (SG). Although SG is growing in favor^2^, some randomized clinical trials have shown less weight loss and smaller improvements in HbA1c or use of more diabetes medications to achieve similar glycemic control compared with RYGB^3–5^.

In addition to restriction of gastric capacity, favorable effects of RYGB on T2DM are thought to result from many factors, including hormonal changes, alterations in gastrointestinal transit time, changes in nutrient absorption and possibly changes in serum bile acids and composition of the microbiome^6^. SG has been less intensively studied but it is believed that many of these factors may also be at play. A major anatomical difference between the two procedures is exclusion of the proximal small intestine with RYGB. Animal studies suggest that duodenal-jejunal bypass improves insulin sensitivity and glucose tolerance independent of changes in body weight, incretin levels, and insulin secretion^7,8^. Another major anatomical difference for the SG procedure is a complete resection of the stomach fundus, which contains most of the ghrelin producing X/A-like cells^9^. Thus, consequences of the different anatomical changes may result in differences in insulin sensitivity and secretion that are independent of weight loss.

The objective of the present study was to delineate changes in beta-cell function after SG in patients with T2DM, and to compare these changes to a RYGB cohort after similar weight loss. An intravenous glucose challenge was utilized in order to isolate the change in beta-cell function from differences in nutrient flow between the two procedures.

## Methods

Subjects with T2DM who were planning to undergo SG (N = 10) and RYGB (N = 10), age 18–75 years, and HbA1c 6.5–12% were recruited. Major exclusion criteria included pregnancy; treatment with glucocorticoids, anti-psychotics, neuroleptics, weight loss medications, or a thiazolidinedione; greater than a 5% change in total body weight in the 90 days prior to enrollment in the study; or triglycerides >400 mg/dL.

An insulin supplemented frequently sampled intravenous glucose tolerance test (fsIVGTT) was performed prior to and after the intervention as previously described^10,11^. Initial weight was defined as the weight obtained at the pre-intervention IVGTT visit. The post-intervention IVGTT was performed once the subject had lost 5–12% of body weight. None of the patients experienced complications during the post-operative period of study. Oral diabetes medications were held 2–3 days prior to testing and no patients were on GLP-1 receptor agonist therapy or insulin. Bergman minimal model analysis (MINMOD Millennium 6.02 software) was used to quantify glucose-dependent glucose elimination (Sg), sensitivity of glucose elimination to insulin (S_i_), acute insulin response to glucose (AIR), and a measure of insulin secretion in relation to insulin sensitivity, the disposition index (DI). Homeostasis model assessment of insulin resistance (HOMA-IR) was calculated as reported^12^. Insulin clearance was calculated using the ratio of fasting C-peptide to insulin^13,14^. Acute C-peptide response (ACPR) was calculated as the relative mean increase (in percent) in C-peptide levels 3–5 minutes after glucose administration.

### Analytic assays

Serum insulin, C-peptide, glucose, total plasma GLP-1, glucagon and total ghrelin were measured as previously described^10,14,15^.

### Statistical Analysis

Power analysis: Based on our prior work^10,16^, nine subjects in each group would provide 80% probability of detecting a difference of 160 in ΔDI with standard deviation (SD) of 120 for SG versus RYGB with a *P* α < 0.05%. The primary endpoint was a comparison of the change in DI between groups. Data are presented as mean ± standard error of the mean (SEM). Group differences in the distribution of continuous variables pre-intervention and between-group differences in change from pre to post-intervention were tested with unpaired Student *t* tests. Within-group differences between pre- and post-intervention were tested with paired Student *t* tests. All *t* tests were two tailed. Two-way repeated measures ANOVA was used to evaluate various parameters for group and time interaction. *P* values < 0.05 were considered statistically significant. Statistical analysis was performed using GraphPad Prism Version 7.02.

Ethical Approval, Informed Consent, and Accordance: The study was approved by the Columbia University Institutional Review Board and written informed consent was obtained from all participants. The methods were carried out in accordance with the Declaration of Helsinki.

## Results

**Figure 1:**
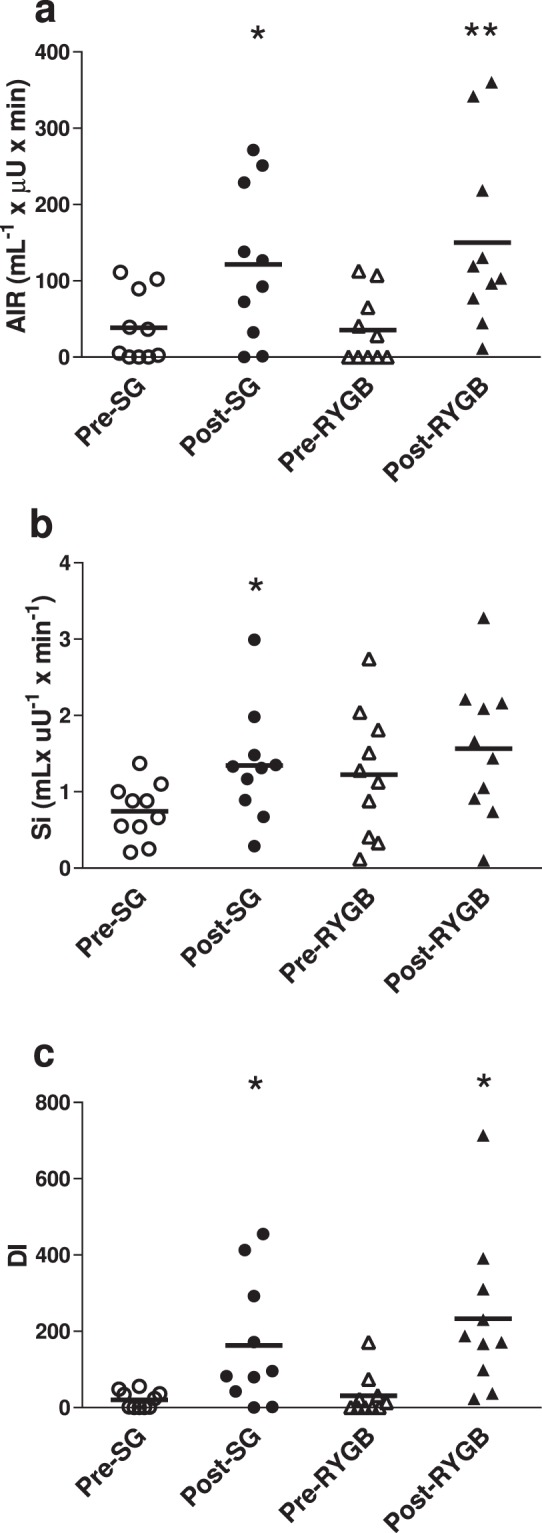
Glucose homeostatic parameters before and after interventions. Plot of individual values. (**a**) AIR; (**b**) Si; (**c**) DI. *p < 0.05, **P < 0.01 within group difference between pre- and post-intervention.

Baseline characteristics including mean age (44.0 ± 2.0 years), BMI (45.2 ± 1.4 kg/m²), HbA1c (7.4% ± 0.2), fasting glucose (152.8 ± 10.2 mg/dL), diabetes duration (4.0 ± 0.9 years), and number of diabetes medications (1.5 ± 0.2) were similar between groups (Table 1). No patients were taking insulin or GLP-1 receptor agonists. Weight loss and change in glucose homeostatic parameters are included in Table 2 and Fig. 1. Mean total weight loss percent between groups was similar (8.4% ± 0.4, p = 0.22) after a period of 21.0 ± 1.7 days. Changes in fasting glucose (p = 0.82), AIR (p = 0.43), and Si (p = 0.47) were not different between groups. Both groups showed increases from baseline to post-surgery in DI (20.2 to 163.3, p = 0.03 for SG, and 31.2 to 232.9, p = 0.02 for RYGB) with no significant difference in the change in DI between groups (p = 0.53). Change in DI did not correlate with percent weight loss for the entire cohort (r = −0.05; p = 0.84), or for the groups considered separately. Data were also analyzed by two-way repeated measures ANOVA and no group x time interaction was detected for glucose (p = 0.82), Si (p = 0.47), AIR (p = 0.43) or DI (p = 0.53) values, although time was significant for all these parameters. Insulin clearance increased to a similar degree post-SG and RYGB (p = 0.94).

**Table 1:** Baseline characteristics.

Parameter	SG	RYGB	P
N (female/male)	10 (6/4)	10 (7/3)	
Age (years)	43.7 ± 2.2	44.3 ± 3.4	0.88
Body weight (kg)	128.0 ± 5.9	128.1 ± 7.4	0.99
BMI (kg/m^2^)	44.2 ± 1.8	46.2 ± 2.3	0.49
Diabetes duration (years)	3.0 ± 1.2	4.9 ± 1.4	0.32
HbA1c (%, mmol/mol)	7.2 ± 0.3 (55.7 ± 3.4)	7.5 ± 0.3 (58.0 ± 3.1)	0.57
Number of diabetes medications	1.5 ± 0.3	1.5 ± 0.2	1.00

Data are presented as mean ± SEM. P-values are shown for between-group comparisons.

**Table 2:** Weight loss and glucose homeostatic parameters before and after surgery.

	SG		RYGB		
	Pre-surgery	Post- surgery	Pre- surgery	Post- surgery	P^a^
Weight loss period (days)		19.2 ± 2.5		22.8 ± 2.3	0.30
Total weight loss (%)		8.9 ± 0.6		7.9 ± 0.6	0.22
Excess weight loss (%)		21.5 ± 1.6		20.1 ± 2.2	0.60
Change in BMI (kg/m^2^)		−3.9 ± 0.3		−3.8 ± 0.4	0.80
Glucose (mg/dL)	156.0 ± 18.4	116.0 ± 12.1	149.7 ± 10.0	114.9 ± 7.7*	0.82
Insulin (uIU/mL)	37.1 ± 12.5	17.7 ± 2.3	24.4 ± 3.6	17.7 ± 4.0*	0.27
C-peptide (ng/mL)	5.3 ± 1.1	3.6 ± 0.3	4.1 ± 0.3	3.6 ± 0.5	0.27
HOMA-IR (mmol × uIU × L^2^)	14.1 ± 4.8	5.2 ± 1.0	8.6 ± 1.2	5.3 ± 1.6*	0.25
Insulin clearance, fasting C-peptide/insulin (ng/uIU)	0.17 ± 0.01	0.21 ± 0.01***	0.20 ± 0.03	0.24 ± 0.03	0.94
Si (mL × uU^−1^ × min^−1^)	0.7 ± 0.1	1.4 ± 0.2*	1.2 ± 0.3	1.6 ± 0.3	0.47
AIR (mL^−1^ × uU × min)	38.6 ± 14.4	121.5 ± 31.9*	35.4 ± 14.3	150.2 ± 37.7**	0.43
Acute C-peptide response (%)	4.0 ± 2.2	20.1 ± 6.8*	7.3 ± 2.6	32.2 ± 2.3***	0.28
Sg (min^−1^)	0.014 ± 0.002	0.011 ± 0.002	0.013 ± 0.003	0.012 ± 0.002	0.22
DI	20.2 ± 7.1	163.3 ± 52.7*	31.2 ± 17.2	232.9 ± 64.4*	0.53

Data are presented as mean ± SEM. P^a^ value for unpaired t-test of between group difference in change from pre- to post-intervention. *P < 0.05, **P < 0.01, ***P < 0.001 within group difference between pre- and post-intervention. Excess Weight Loss (%) calculated using the calculation of ideal body weight as that equivalent to a BMI of 25 kg/m^2^.

Changes in plasma hormone levels are presented in Table 3. Ghrelin levels decreased significantly post-op in the SG group but no change was observed after RYGB and there was a significant group difference in the change in ghrelin levels (p < 0.001). GLP-1 levels increased after RYGB (p = 0.07) and decreased after SG (p = 0.06), and the difference in the change in GLP-1 levels between groups was significant (p = 0.01). However, the insulin:GLP-1 molar ratio was also calculated and a similar decrease in this ratio post-SG and RYGB was observed (p = 0.41). There was no significant change in PYY in either group. Glucagon levels decreased to a significant degree post-SG whereas a non-significant increase was observed post-RYGB and the difference in the change in glucagon levels between groups was significant (p = 0.03). However, the change in the insulin:glucagon molar ratio did not differ between groups.

**Table 3:** Fasting plasma hormone levels before and after surgery.

	SG		RYGB		
	Pre-surgery	Post-surgery	Pre-surgery	Post-surgery	P^a^
GLP-1 (pmol/L)	22.8 ± 2.3	19.4 ± 2.1	9.3 ± 1.9	14.3 ± 3.5	0.01
Insulin:GLP1 molar ratio	14.6 ± 5.9	7.9 ± 1.8	23.1 ± 5.0	12.0 ± 3.2*	0.41
PYY (pg/mL)	86.5 ± 23.5	72.0 ± 19.2	79.0 ± 22.8	71.6 ± 17.0	0.76
Ghrelin (pg/mL)	371.7 ± 37.4	111.1 ± 8.4***	245.5 ± 18.6	256.4 ± 26.1	<0.001
Glucagon (pg/mL)	101.5 ± 11.8	77.3 ± 6.0*	79.0 ± 11.0	86.4 ± 15.0	0.03
Insulin:Glucagon molar ratio	9.4 ± 2.6	6.0 ± 0.8	7.7 ± 1.1	4.8 ± 1.0*	0.82

Data are presented as mean ± SEM. P^a^ value for unpaired t-test of between group difference in change from pre- to post-intervention. *P < 0.05, **P < 0.01, ***P < 0.001 within group difference between pre- and post-intervention. Samples for measurement of plasma ghrelin were unavailable for 2 RYGB subjects. Outliers identified by ROUT (Q = 1%) were removed for GLP-1 values for 1 subject in each group.

## Discussion

This study demonstrates that SG results in a similar short-term improvement in beta-cell function compared with RYGB in subjects with T2DM. After a similar reduction in body weight achieved over the same period of time, improvement in DI was nearly identical between the two procedures. Differences in the change in secondary outcomes, including fasting glucose, insulin sensitivity, and insulin secretion were also not observed. These results suggest that the anatomical differences between the two procedures do not modify the intrinsic improvement in beta-cell function at this early post-operative period although it is important to note that an intravenous instead of an oral nutrient challenge was utilized in order to eliminate potential confounding factors related to altered nutrient flow.

Our results are in agreement with a study by Bradley *et al*. that demonstrated similar improvements in insulin sensitivity and beta-cell function using a hyperinsulinemic–euglycemic clamp procedure after a matched 20% weight loss in subjects without T2DM^17^. In contrast, using a hyperglycemic clamp, Kashyap *et al*. compared RYGB with laparoscopic adjustable band (LAGB) and SG in patients with obesity and T2DM at one and four weeks after surgery and found that insulin sensitivity improved only in the RYGB group^18^. However, these results are difficult to interpret given that SG was not studied independent of LAGB, which has been shown to be a somewhat inferior metabolic surgery for the treatment of T2DM^19^.

Basso *et al*. evaluated very early changes in glycemic parameters and gut hormones in subjects with obesity and T2DM using an IVGTT three days after SG and found a significant increase in insulin secretion and a significant improvement in peripheral insulin sensitivity in subjects with diabetes for less than 10.5 years^20^. A reduction in ghrelin, increase in GLP-1 (both basal and 15 minutes after glucose infusion), and increase in glucose-stimulated PYY post-op were observed after SG. The authors proposed a “gastric hypothesis” in which an extra-pancreatic and/or extra-intestinal factor that inhibits insulin secretion may be produced in the stomach and excision of the gastric fundus in SG removes this “factor” and improves insulin secretion. In our study, there is no additional evidence of a negative gastric factor (anti-incretin or anti-insulin sensitivity factor) with IV stimulus or in the fasting state as the results for RYGB and SG were similar.

Changes in insulin sensitivity and beta-cell function have been compared after RYGB and SG using an oral meal challenge. In agreement with our findings, Wallenius *et al*. found similar improvements in glucose control and insulin secretion using a modified 30 g oral glucose tolerance test (MOGTT) 2 days, 3 weeks, and 1 year after SG and RYGB, despite a significantly greater weight loss and increase in GLP-1 with RYGB at 1 year^21^. At 15 days after RYGB or SG, Nannipieri *et al*. found that both SG and RYGB groups showed similar increased insulin secretion rates and modest improvements in beta-cell sensitivity^22^. At 1 year, after marked weight loss, which was somewhat more after RYGB (p = 0.09), similar improvements in glycemic control, diabetes remission, insulin sensitivity, and beta-cell glucose sensitivity were observed. Romero *et al*. reported that at 4–6 weeks post-surgery with similar weight loss, both SG and RYGB groups showed similar improvements in glucose tolerance and DI^23^. At two years post-op in a metabolic substudy of the STAMPEDE trial, Kashyap *et al*. reported marked improvements in beta-cell function (oral DI) after RYGB and only negligible improvement after SG with similar weight loss^24^. However, there was not a statistical difference between groups in change in DI (p = 0.34) or change in insulin sensitivity (p = 0.39).

Our results indicate a similar improvement in fasting hepatic insulin clearance post- RYGB and SG, suggesting the liver plays a role in the early improvement in glycemic control. Our findings are in agreement with Immonen *et al*. who demonstrated improvements in several hepatic glycemic parameters, including insulin clearance in a combined SG and RYGB group at 6 months post-op^25^.

We cannot exclude the possibility that factors unique to each procedure result in independent mechanisms by which improvements in glycemic control are achieved, particularly after a nutrient challenge. In RYGB, exclusion of the proximal small intestine may result in a greater improvement in insulin secretion due to the nutrient exclusion from the proximal small intestine and rapid delivery of unabsorbed nutrients to the distal small intestine which is thought to enhance the secretion of GLP-1 and PYY^6^, whereas the role of GLP-1 after SG has been less well studied^26^. There is evidence of accelerated gastric emptying after SG shown in scintigraphy studies^27^, which may be followed by delayed small intestinal transit. These factors may conceivably enhance postprandial GLP-1 and PYY release after SG as well. In the present study, fasting levels of GLP-1 and PYY did not change significantly post-SG or RYGB; however we did not assess post-prandial glucose or gut hormone levels given our experience of patients feeling ill after a significant oral nutrient load in this early post-operative period.

Due to the resection of the gastric fundus, fasting ghrelin levels post-SG were significantly reduced, whereas there was no change post-RYGB. It appears this preferential decline in ghrelin post-SG is durable out to at least 18 months^28^. The reduction in ghrelin levels after SG may enhance insulin secretion^29^ and insulin sensitivity^30^. A reduction in glucagon was observed only post-SG. However, the insulin:glucagon molar ratio decreased to a similar extent in both groups, suggesting a relative hyperglucagonemia after both surgeries. In the Diabetes Surgery Study, we found a similar change in the insulin:glucagon molar ratio after RYGB, but no significant change after intensive medical management^14^. The relative hyperglucagonemia after both SG and RYGB may be important for the metabolic benefits^31^.

It is also likely that in the longer-term RYGB may provide additional benefits compared with SG as was shown in the STAMPEDE trial in which there was greater weight loss and an equivalent improvement in HbA1c achieved after 5 years with the use of fewer diabetes medications^5^. The extent to which differences in weight loss impact the different outcomes seen with these procedures is unclear. We have previously shown that improvement in HbA1c correlates with the percent weight loss one year after RYGB^14^. In this study, there was no correlation between change in DI and weight loss, however, the range of weight loss was likely too narrow and the profound caloric restriction at this early time-point may supersede the ability to detect the weight loss effect.

A major strength of this study is that groups were well matched not just for baseline characteristics, but importantly, they were also matched for weight loss and duration of the post-operative period of study. However, limitations of this study include the nonrandomized design, the relatively small sample size and assessment of only short-term changes. Additionally, we did not assess post-prandial glucose or gut hormone levels. While it has been shown that there are important changes in fasting gut peptide levels after SG and RYGB^20,32–34^, it is important to acknowledge that results might differ if beta-cell function and gut hormone changes were assessed using an oral nutrient challenge. Finally, our study did not include a diet-induced weight loss control group and caloric restriction is known to independently improve glycemic control^35^. At the post-operative study visit, SG and RYGB subjects were on a pureed diet of approximately 500 kcal/day and this degree of caloric restriction likely plays a role in the observed early improvements in diabetes. We did compare the SG group to a similar cohort of subjects with diabetes placed on a very low calorie diet as previously reported^10^ (data not shown) and similar improvements in beta-cell function were also observed. Our findings suggest that some of the early improvements in diabetes in both the SG and RYGB groups may be a consequence of caloric restriction and weight loss, rather than mechanisms unique to a bariatric procedure. However, from several randomized controlled clinical trials it has become clear that clinically surgery produces greater and more durable improvements in glycemic control^4,5,36,37^. We cannot determine from this study whether differences between these procedures would be observed if subjects were studied at equivalent weight loss at a later post-operative period once caloric intake is liberalized.

## Conclusions

SG improves beta-cell function as well as RYGB in the short term in obese patients with T2DM when assessed using an IV glucose challenge. However, further studies are needed to determine if longer-term clinical outcomes tend to be better after RYGB due to greater weight loss and/or other factors that differ between the procedures as a consequence of the different anatomical alterations.

## Data Availability

The datasets generated and analyzed during this study are available from the corresponding author on reasonable request.
